# Three-dimensional cellular aggregates formed by *Beauveria pseudobassiana* in liquid culture with potential for use as a biocontrol agent of the African black beetle (*Heteronychus arator*)

**DOI:** 10.1080/21501203.2020.1754953

**Published:** 2020-04-27

**Authors:** Laura F. Villamizar, Gloria Barrera, Sean D.G. Marshall, Marina Richena, Duane Harland, Trevor A. Jackson

**Affiliations:** a Lincoln Research Centre, AgResearch Ltd, Christchurch, New Zealand; b Control Biológico De Plagas Agrícolas, Colombian Corporation for Agricultural Research, Vía Mosquera, Colombia

**Keywords:** Cell aggregate, *Beauveria*, entomopathogenic fungus, biocontrol, African black beetle, *Heteronychus arator*

## Abstract

*Beauveria pseudobassiana* formed three-dimensional aggregates of cells (CAs) in liquid culture. CAs were formed mainly by blastospores and conidia, distinct from microsclerotia formed through adhesion of hyphae. The formation, germination and sporulation of CAs were studied, as well as the pathogenicity of conidia produced from them against adults of black beetle. After 4 days of culture, CAs were formed, becoming compact and melanised after 10 days of incubation. Electron microscopy showed three-dimensional CAs averaging 431.65 µm in length with irregular shapes and rough surfaces, where cells were trapped within an extracellular matrix. CAs germinated after 2 days of incubation on agar-plates producing hyphae and forming phialides and conidia after 4 days. Produced conidia caused 45% mortality of black beetle adults. CAs germination and sporulation on soil were directly correlated with soil moisture, reaching 80% and 100% germination on the surface of soil with 17% and 30% moisture, respectively. CAs maintained 100% germination after 2 years of storage under refrigeration. These CAs could have a similar function as microsclerotia in nature, acting as resistant structures able to protect internal cells and their ability to sporulate producing infective conidia, suggesting their potential to be used as bioinsecticides to control soil-dwelling insects.

## 1. Introduction

Critical constraints to the commercial development of a microbial biocontrol agent include the ability of the microorganism to form a robust, stable, infective propagule and the development of a cost-effective method for massive production (Ravensberg 2011). For entomopathogenic fungi, this generally involves production of conidia through solid-state culture (SSC) (Jaronski and Mascarin 2016). SSC has proven adaptable to many species and strains of entomopathogenic fungi, but the method usually involves small batch cultures with associated problems of consistency and scalability of production. Poor storage stability of the resultant conidia and poor persistence in the environment after application have been identified as weaknesses in SSC produced products.

Liquid fermentation is an alternative production method for entomopathogenic fungi that is scalable and can produce large volumes of a uniform product. Mycelia, blastospores (BS) and submerged conidiospores (SCS) can be cultured in liquid fermentations to produce high yields using state-of-the-art process control (Lohse et al. 2014). However, some of these propagules are less tolerant to desiccation and storage in comparison with SSC produced conidia (Chong-Rodríguez et al. 2011).

Recently it was discovered that, under certain conditions, entomopathogenic fungi can form microsclerotia that can survive long periods in adverse environments and have the potential to be developed as robust fungal products (Jaronski and Jackson 2009; Song et al. 2014). Microsclerotia (MS) have been traditionally described for many fungal plant pathogens as small (100–1000 µm), multicelled and melanised mycelia aggregates, that allow infective propagules to survive for long periods in the soil (Soesanto and Termorshuizen 2001; Varo et al. 2016). MS formation in liquid fermentation of entomopathogenic fungi was first described for *M. anisopliae* (Jaronski and Jackson 2008). Different strains of this fungus have been able to form MS in shaking flask cultures using media with varying carbon concentrations and carbon-to-nitrogen (C:N) ratios and the structures have been consistently described as compact hyphal aggregates that become pigmented with culture age (Jackson and Jaronski 2009). Subsequently, the formation of similar structures has been reported for other biocontrol agents such as *Trichoderma harzianum* (Kobori et al. 2015), *M. brunneum* (Jaronski and Jackson 2012), *M. rileyi* (Song et al. 2017) and *Paeilomyces lilacinum* (Song et al. 2016).

The formation of MS in *Beauveria* species was reported for the first time from *B. bassiana* by Wang et al. (2011), who described the formation of melanised aggregates of fungus. Subsequently, Villamizar et al. (2018) reported MS formation in three species of this genus, where the MS were described as originating from aggregations of hyphae of variable size and shape. Recently, Huarte‐Bonnet et al. (2019) described microsclerotia-like pellets formed by *B. bassiana*, which consisted of compact hyphal aggregates found in 4-day-old cultures with production mediated by oxidative stress and peroxisome biogenesis.

Fungi can also form biofilms, which are aggregated communities encased in a protective extracellular matrix (Donlan 2001), which has been described for several clinically relevant fungi such as *Candida* spp., *Aspergillus* spp., *Cryptococcus neoformans, Fusarium* spp., *Blastoschizomyces capitatus, Malassezia pachydermatis, Pneumocystis* spp., *Trichosporon asahii, Rhizopus* spp., and *Rhizomucor* spp (Kernien et al. 2018). Filamentous fungal biofilms have been poorly reported, possibly because filamentous fungi generally do not fit completely or precisely within the restrictive biofilm definitions that are based on bacterial and yeast models (Peiqian et al. 2014).

Fungal biofilms are mainly defined as heterogeneous, surface-associated colonies comprised of filamentous hyphae, pseudohyphal cells, yeast-form cells, and various forms of extracellular matrix (Lagree et al. 2018). These structures have distinct developmental phases, including adhesion, colonisation, maturation and dispersal, which are governed by complex molecular events. Biofilms possess survival advantages in harsh environments and resistance to chemical and physical detrimental factors (Ramage et al. 2009), which has been related with the self-produced extracellular matrix composed of proteins, extracellular DNA, lipids, and mono- and polysaccharides (Sheppard and Howell 2016)

There has been a growing interest in the use of entomopathogenic fungi for the control of insect pests. In this study, we tested *B. pseudobassiana* against adults of the African black beetle, *Heteronychus arator* (Fabricius 1775) (Coleoptera: Scarabaeidae), an important pasture pest in New Zealand (Mansfield et al. 2016; Ferguson et al. 2019).

To effectively control the soil-dwelling *H. arator*, a fungal biopesticide applied to soil must be able to create a persistent zone of infectious conidia through which the target insects must pass and acquire enough conidia through physical contact to result in infection. Although conidia can be applied to soil in aqueous suspensions, application with standard farm equipment to achieve targeted placement and homogenous distribution through the soil matrix can be difficult (Jaronski and Jackson 2008). In this context, a granular formulation is more practical and the key principle would be to use an active ingredient based on, or capable of producing, robust, stable, persistent and infective propagules.

Even though *Beauveria* spp. are some of the most studied and commercially applied entomopathogenic fungi, there is a lack of information addressing the formation of aggregate structures in this genus and their potential to be developed as biopesticides. In this study, we describe the formation process of three-dimensional cell-aggregates produced by a New Zealand *B. pseudobassiana* isolate obtained from *H. arator*. We studied the germination and sporulation of these propagules and demonstrated the pathogenicity of produced conidia against adults of the African black beetle.

## 2. Materials and methods

### 2.1 Cultures

The *B. pseudobassiana* isolate AgR-F704 used for this study was obtained from the AgResearch fungal culture collection. The isolate was grown on potato dextrose agar (PDA) (Merck), and incubated for 15 days at 25°C. Conidia were harvested by rinsing the plates with 10 mL 0.01% Triton X-100 and recovered conidia were subsequently used to inoculate culture flasks.

### 2.2 Production of three-dimensional aggregates in liquid culture

Liquid culturing was conducted in non-baffled 250 mL conical flasks containing 100 mL of culture medium according to the composition described by Villamizar et al. (2018). The medium incorporated a mixture of salts with the addition of trace elements (mineral solution), but without vitamins (Jaronski and Jackson 2009), and supplemented with acid-hydrolysed casein (Becton Dickson) and corn steep liquor (CSL; NZ Starch Ltd). The medium contained (per litre): 1.0 g KH_2_PO_4_, 0.2 g CaCl_2_⋅2H_2_O, 0.15 g MgSO_4_⋅7H_2_O, 35 mg ZnSO_4_⋅7H_2_O, 25 mg FeSO_4_⋅7H_2_O, 9 mg CoCl_2_⋅6H_2_O, 4 mg MnSO_4_⋅H_2_O, 45 g casamino acids, and 75 g CSL. Because of the high viscosity of CSL and its tendency to clump when autoclaved, a stock solution in deionised water was prepared and autoclaved separately (150 g/L). The final medium was prepared by mixing 50 mL of a 2x mineral solution with 50 mL of the 2x CSL solution (previously filtered through a 100 μm sterile wire mesh). Three flasks were inoculated with 1 mL of a 10^6^ conidia/mL spore suspension and incubated at 25°C and 300 rpm on a benchtop rotary shaker (Infors HT Ecotron). Each flask was vigorously hand-shaken once daily for the first 5 days to minimise the growth of mycelia on the sides of the flasks; thereafter any mycelial rings that formed on the side of the flasks were aseptically removed at subsequent sampling times. On days 2, 4, 7 and 10, one mL samples of whole culture broth were aseptically collected. To follow the fungal development and check for cell-aggregates (CA) formation, 100 µL of culture broth was placed on a glass slide and gently overlaid with a 22 × 50 cm glass coverslip. Microscopic observations were made using phase contrast optics with an Olympus BX-50 microscope and photos captured using an Olympus DP72 digital camera. When CAs were observed, the number of them was counted across the entire coverslip area.

To determine the nature of the cells in the CAs, a hydrophobicity test was carried out (Boucias et al. 1988) by harvesting some structures that were then washed twice in distilled water to remove free cells. Three samples of clean CAs were independently suspended in 500 µL of distilled water and completely disrupted in a Eppendorf tube by using a pestle. Cell concentration in the suspensions was quantified using a Neubauer haemocytometer and then adjusted to 1 × 10^7^ cells/mL. Samples (300 µL) of each suspension were poured into 1.5 mL Eppendorf tubes and mixed with equal volumes of xylol (organic phase) by vertically inverting the tube 10 times. Tubes were incubated under refrigeration overnight to allow the phases to separate and cell concentration was again determined in the aqueous phases by counting in Neubauer haemocytometer.

### 2.3 Harvesting of AgR-F704 aggregates

Three-dimensional aggregates/biofilms were harvested from 10-day-old cultures by adding 5 g of diatomaceous earth (DE) Celite® 281 to each 100 mL of culture broth, and then vacuum-filtering in a Buchner funnel through Whatman No. 1 filter paper to remove spent media (Kobori et al. 2015). The resulting filter cake was manually granulated using 1 mm mesh, layered in Petri dish plates, and airdried overnight under laminar flow at 22°C. 250 mg samples were stored at 4°C in microcentrifuge tubes (2 mL) sealed with parafilm.

### 2.4 Description of germination and sporulation of AgR-F704 aggregates

Viability of CAs was measured immediately after drying and after 24 months of storage at 4°C by determining the presence of hyphal growth after 48 h of incubation (germination). Dry granules containing cell-aggregates (250 mg) were resuspended in 1 mL of 0.05% Tween® 80 (Sigma) with 100 μL samples inoculated onto three water agar plates (1.5% agar w/v) using spread plate techniques and incubated at 25°C. Agar plates were evaluated by observation through a stereomicroscope (Olympus SZX12). The total number of CAs and the number of CAs displaying hyphal growth were recorded and germination rates were then calculated as a percentage ratio.

Another three water agar plates were inoculated following the same methodology to study the germination process. CAs were recovered immediately after plate inoculation and after 48 and 72 h of incubation, and were processed for transmission and scanning electron microscopy (TEM and SEM) observations. To protect the structure during processing for TEM, samples were embedded in an agarose matrix (0.5% w/v), which was immersed in glutaraldehyde 2.5% prepared in buffer phosphate (pH 7.4) for 12 h at 4°C. Samples were then post-fixed in osmium tetraoxide (1%) for 2 h and dehydrated with ethanol in ascendant concentrations. Finally, the samples were embedded in acrylic resin (LR white medium grade) and polymerised at 60°C. The blocks were thin sectioned (40 nm) and contrasted with uranyl acetate and lead citrate as described previously (Reynolds 1963). The samples were observed in an Electron Microscopy (JEOL 1400 plus). For SEM, CAs were directly fixed with glutaraldehyde 2.5% (pH 7.4) and dehydrated with ethanol in ascendant concentrations. The samples were sputtering with colloidal gold and observed in an electron microscopy (JEOL JSM 7000 F).

### 2.5 Germination and sporulation of AgR-F704 aggregates on soil

The soil (50 g) for assays was pasteurised by microwaving for 2 min at maximum power and then dried overnight in a pre-heated oven at 100°C. The dry soil was divided into two 25 g samples and moisture was adjusted to 17% and 30% with sterile distilled water. Three wells of two columns of a 24-wells plate were filled with 1 g of soil at 17% moisture and the same process was carried out in the next two columns with soil at 30% moisture. Each column corresponded to one treatment with three experimental units (three wells). Ten three-dimensional aggregates/biofilms were placed on the surface of the soil contained in each well of the first column for each soil moisture. The second column of wells containing soil was not inoculated and corresponded to the control treatment. The inoculated plate was incubated at 25°C for 48 h and germination was assessed by determining the presence of hyphal growth. All CAs were observed through a stereomicroscope (Olympus SZX12), to record the total number of structures per well and the number of them displaying hyphal growth. Germination rates were calculated as a percentage ratio, with plates then incubated for an additional 5 days at 25°C to assess conidia production. To measure conidia production, the complete content of each well was recovered with 9 mL sterile 0.05% Tween® 80 (Sigma). Subsequently, serial 10-fold dilutions were performed and 100 μL aliquots of the 10^−3^ and 10^−4^ dilutions from each sample were plated in triplicate onto Rose Bengal Chloramphenicol Agar (Thermo Scientific™ Oxoid™). Soil from control treatments were submitted to the same protocol to establish the baseline of *Beauveria* sp. population in utilised soil. After 7 days of incubation, *Beauveria*-like colonies were counted and results expressed as CFU/g of soil. The experiment was repeated twice.

### 2.6 Pathogenicity of conidia produced by AgR-F704 aggregates against black beetle

Granules containing CAs (250 mg) were resuspended in 1 mL of 0.05% Tween® 80 (Sigma) with 100 μL samples inoculated onto water agar plates (1.5% agar w/v) and incubated at 25°C for 7 days. Conidia produced after sporulation of CAs were recovered by rinsing the agar surface with 0.10% Tween® 80. The conidia suspension was then gently probed with a pipette tip, quantified with a Neubauer chamber and adjusted to 1 × 10^6^ conidia/mL. Conidial viability was quantified by spreading a 100 μL aliquot onto three Petri dishes containing water agar and incubated overnight at 25°C. Plates were examined to determine whether or not conidia were viable, as indicated by the presence of germ tubes and expressing the result as germination percentage (Grijalba et al. 2018).

Black beetle adults collected in the field were maintained for a week to assess health and then used for the bioassay. Three batches of 15 beetles were inoculated by gently shaking them in 15 mL of a conidia suspension (1 x 10^6^ conidia/mL) for 30 seconds. The same procedure was followed for the control treatment but using distilled water. Beetles were then individually placed into separate wells of a 24-wells plate and the plates were incubated at 25°C. Pieces of fresh carrot were regularly provided as food, and mortality was evaluated after 15 days.

The Schneider-Orelli formula was used for calculating efficacy (Zar 1999).

$$Efficacy\left(\%\right)=\frac{\left(A-C\right)}{\left(100-C\right)}x100$$

where,

A = Mortality in the treatment

C = Mortality in the control treatment

### 2.7 Statistical analysis

Experimental designs utilised complete randomised blocks with three replicates per treatment. Mean values of germination and insect mortality were compared by one-way ANOVA and Least Significant Difference (LSD) test (95%) using Statistix 8.1 (Analytical Software, Tallahassee, FL, USA).

## 3. Results

### 3.1 Formation of three-dimensional aggregates in liquid culture

**Figure 1.**
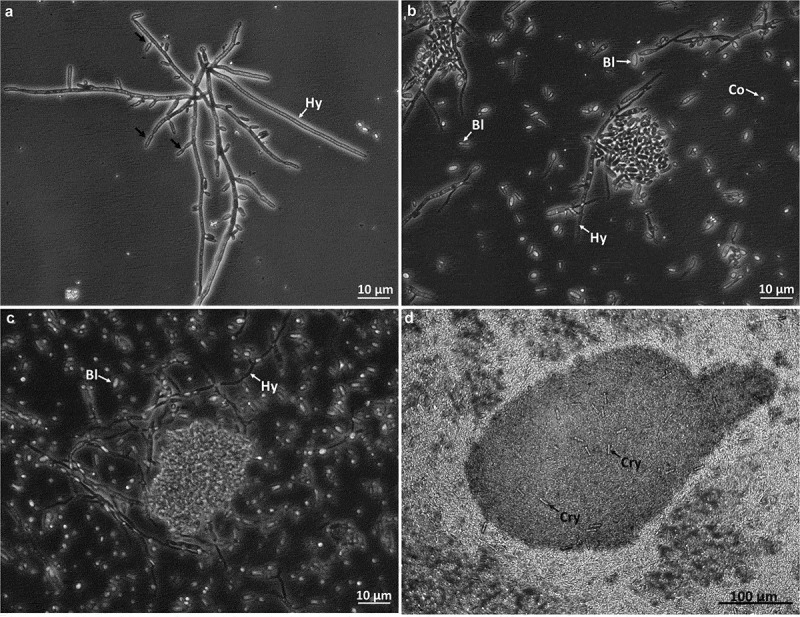
Photographs of CAs development from *B. pseudobassiana* (AgR-F704) in liquid cultures at days 2 (A), 4 (B), 7 (C) and 10 (D) of incubation. Hypha (Hy), Blastospore (Bl), Conidium (Co), Crystal (Cry) and black arrows showing the formation of blastospores

Ungerminated and germinated conidia, incipient hyphal aggregates and forming blastospores were found in 2-day-old cultures (Figure 1a). By day 4, abundant free blastospores (swollen ellipsoidal to cylindrical cells with a dimension 2–4 µm x 1–2 µm) and few conidia (spherical with diameter around 1–2 µm) were observed and aggregates of them were forming attached to polar hyphae (Figure 1b). By day 7, larger, more organised and compact cell-aggregates were observed as well as high concentration of blastospores and conidia (Figure 1c). After 10 days of culturing cell-aggregates reached diameters between 225 and 632 µm (Figure 1d) and appeared compact, with some exhibiting strong melanisation and reaching a yield of 7.54 ± 2.30 x 10^3^ CAs/mL. Abundant blastospores, conidia and free hyphae were still produced and remained as free propagules distributed in the broth. Crystalline structures were observed freely distributed in the broth and trapped withing the CAs (Figure 1d).

The hydrophobicity test showed that after 18 hours of extraction with the organic phase, the initial concentration of free cells released from the CAs was reduced from 1 × 10^7^ cells/mL to 4.9 × 10^6^ cell/mL, indicating that 51.33% of cells (±14.59) were excluded from the aqueous phase. Excluded cells correspond to those with hydrophobic affinity, possibly submerged conidia (either hydrophobic and hydrophilic). Most of the cells remaining in the aqueous phase were elongated and ellipsoidal, consistent with blastosphores that are predominantly hydrophilic (Holder and Keyhani 2005).

### 3.2 Description of germination and sporulation of AgR-F704 aggregates

Immediately after harvest, CAs were of irregular shape, with rough surfaces which turned a light brown colour after drying. All CAs from samples inoculated in water agar immediately after granules drying and after 24 months of storage at 4°C were able to germinate, developing mycelia growth that completely covered the surface after 48 h incubation.

**Figure 2.**
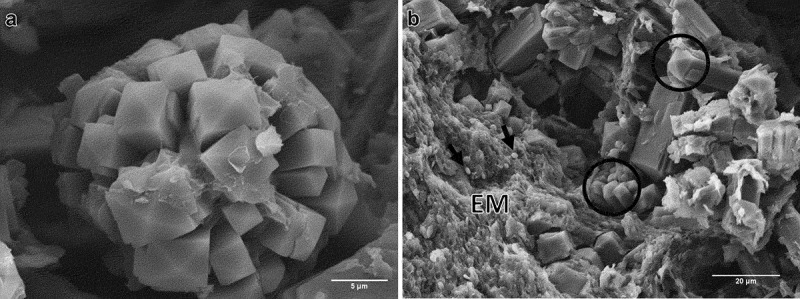
Micrographs of fresh CA produced by *B. pseudobassiana* AgR-704. A. Scanning electron micrograph of agglomerated tetragonal pyramidal crystals inside the CA. B. Scanning electron micrograph of structures inside the CA. Circles show prismatic crystals. Arrows indicate ellipsoidal cells, presumably blastospores. Extracellular matrix (EM)

The crystalline structures previously observed during the CA formation were found trapped in the structure of dry CAs when analysed under scanning electron microscopy. Crystalline structures presented tetragonal pyramidal crystals (Figure 2a and 2b), with some organised in agglomerates (Figure 2b).

**Figure 3.**
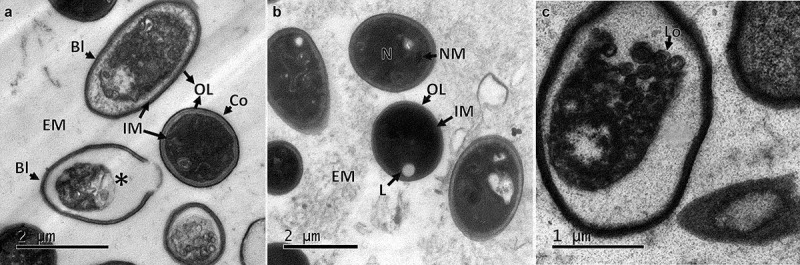
Ultrathin sections of CAs produced by *B. pseudobassiana*. A. Internal ultrathin section showing variable shapes and sizes of cells, corresponding to blastospores (Bl) and Conidia (Co) with outer layer (OL), internal membrane (IM) and extracellular matrix (EM). Asterisc shows the last phase of lysis of a cell. B. Submerged conidia showing nuclear membrane (NM), OL, IM and Lipid (L) surrounded by EM. C. Cell containing membranous structures corresponding to Lomasomes (Lo)

Observation of ultrathin sections of the CAs by TEM indicated the absence of any membrane covering the structure or any particular formation of cells on the edge. The CA was filled with cells with similar cell density in the centre and edges, indicating no difference in the localisation of cells within the structure. An electron-dense material similar to a mucilaginous matrix was observed in the intercellular space. Although the composition has not yet been determined, this material seems to play a role in promoting and maintaining the agglomeration of cells in order to produce the compact structure (Figure 3). The cells forming the CAs were of irregular shape, some were spherical ranging between 1 and 2 µm (conidia) and some were ellipsoidal with 2–4 µm x 1–2 µm (blastospores), filled with electron-dense pigment without defined organelles. The external membranes of blastospores and submerged conidia showed differences between them, with thinner outer layers found in blastospores in comparison with conidia (Figure 3a). Some cells contained autophagic vacuoles or empty cytoplasm indicating a process of autolysis (Figure 3a). In some cells, lipid deposits (Figure 3b) and intracytoplasmic membranous structures (Figure 3c) appeared similar to membranosomes or lomasomes described by Weisberg and Turian (1974) and Wilsenach and Kessel (1965).

**Figure 4.**
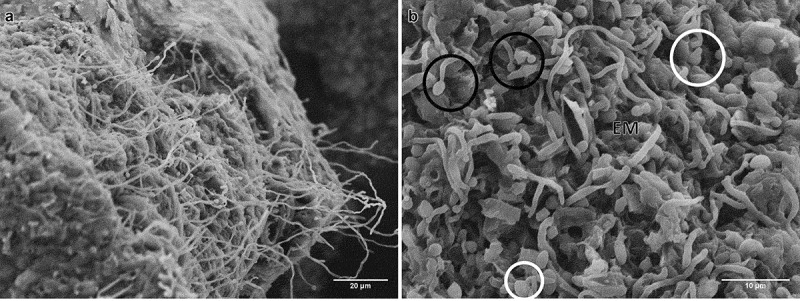
Scanning electron micrographs of CAs produced by *B. pseudobassiana* after 24 h postincubation on water agar plates. A. CA surface with hyphae growth. B. Hyphal bodies emerging from CAs showing germ tube formation (white circles), conidiogenus cells (black circles) and extracellular matrix (EM)

After 24 h of incubation on water agar plates, CAs germinated forming hyphae and mycelia on the surface (Figure 4a); germinating blastospores with different length germ tubes were observed over the entire surface (Figure 4b).

**Figure 5.**
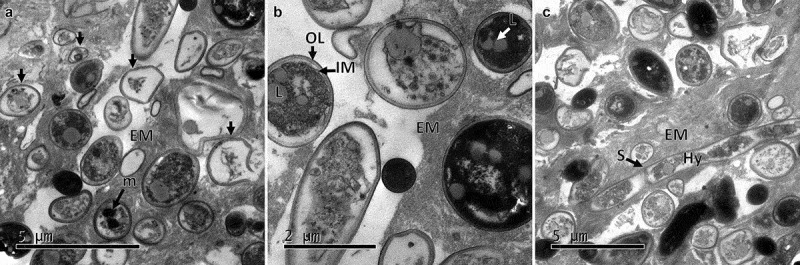
Transmission electron micrographs of CAs produced by *B. pseudobassiana* after 24 h post-incubation on water agar plates. A. CA ultrathin section showing numerous cells in lytic process and dead cells (arrows). B. CA cells with heavy deposits of melanising material. C. CA ultrathin section showing hyphae (Hy) with septums (S). Outer layer (OL), internal membrane (IM), extracellular matrix (EM), lipid (L), melanin deposit (m)

Internally the CAs showed an increase of cells undergoing autolysis. The extracellular matrix increased the electrodensity and became a more fibrillar structure. The cells swelled (Figure 5a) exhibiting a larger size in comparison with the cells observed in the dry structures (Figure 3). A greater number of cells in the process of autolysis were observed with autophagic vacuoles and cells with melanin deposits seen throughout the cytoplasm (Figure 5b). Some cells formed septate hyphae or germinated conidium surrounded by melanised cells.

**Figure 6.**
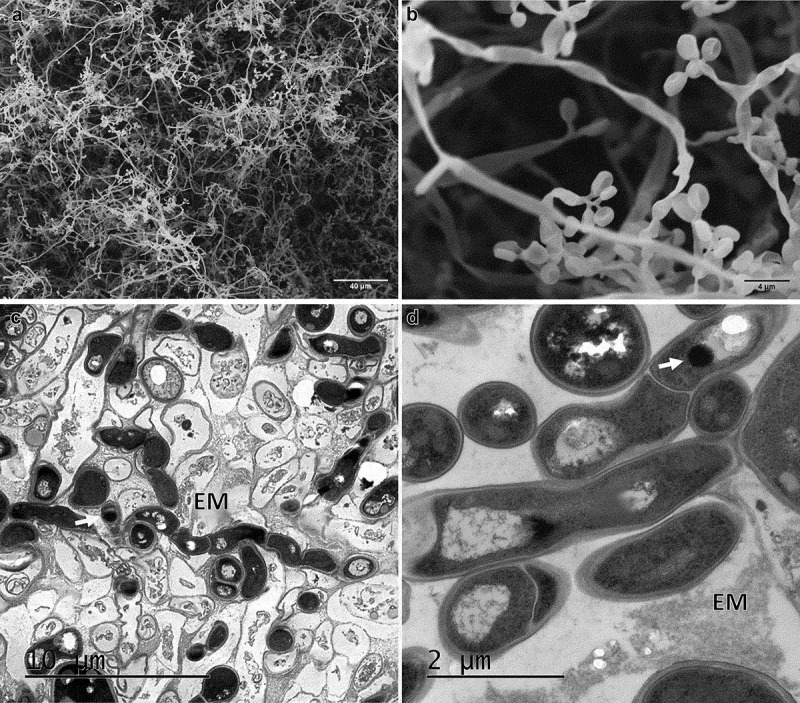
Electron micrographs of CAs produced by *B. pseudobassiana* after 72 h post-incubation in water agar. A and B Scanning electron micrographs of CAs showing mycelium and phialide details. C and D. Ultrathin sections of hyphae division with melanin deposits (white arrows)

After 72 h in water agar, CAs formed interwoven hyphae on the surface (mycelium) with several phialidic conidiogenous cells (Figure 6a,b). The ultrathin sections showed several hyphae (Figure 6c,d) surrounded by dead cells. Melanin deposits were observed inside the hyphae.

### 3.3 Germination and sporulation of AgR-F704 aggregates on soil

**Figure 7.**
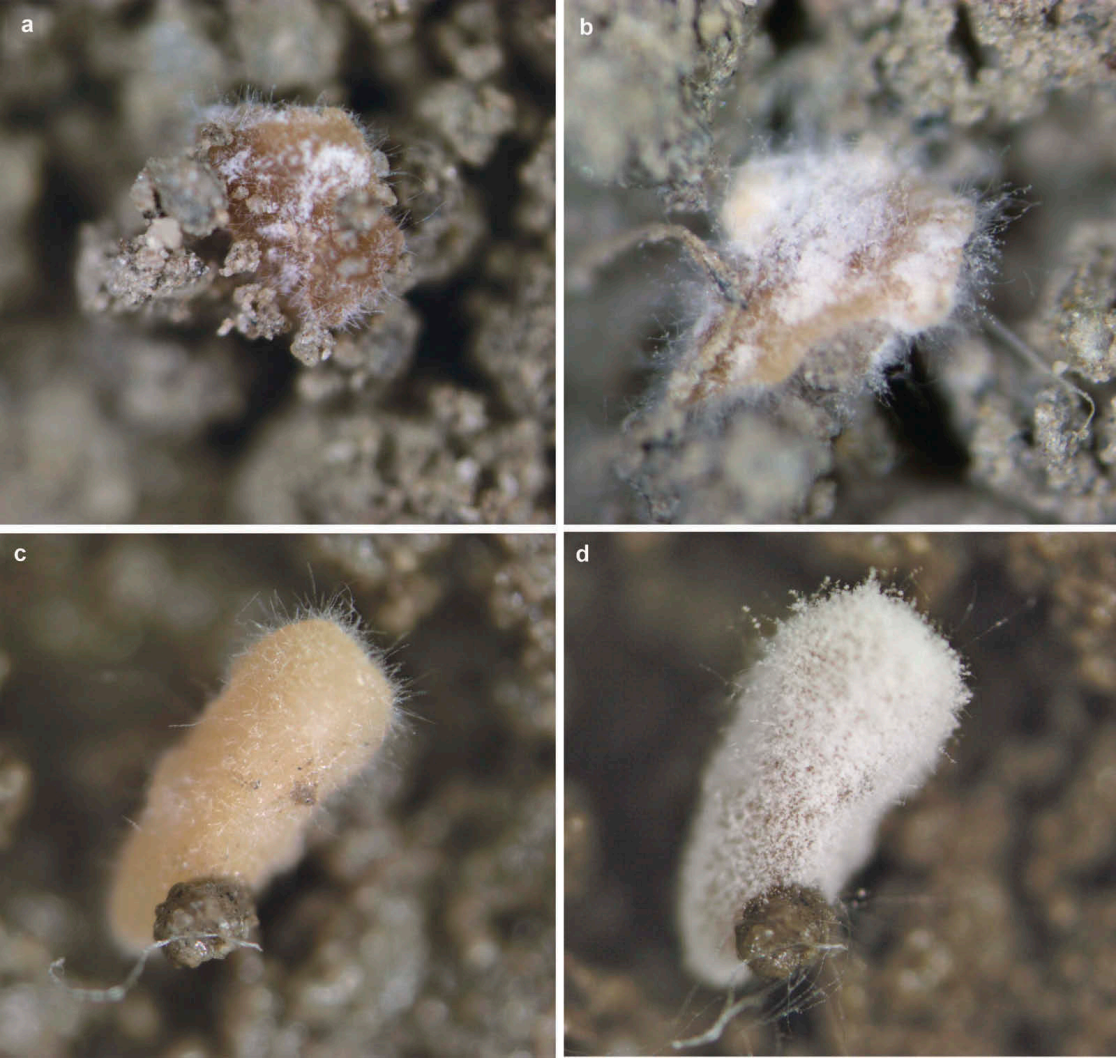
Germination of CAs produced by *B. pseudobassiana* on soil with different moisture content. (A) 48 h on soil at 17% moisture, (B) 7 days on soil at 17% moisture, (C) 48 h on soil at 30% moisture, (D) 7 days on soil at 30% moisture

The CAs germinated after 48 h of incubation on the soil surface with two different moisture levels, forming hyphae and mycelium on the surface of the structures. Sporogenic germination was observed after 7 days for fungal treatments at both moisture levels (80% and 100% germination at 17% and 30% soil moisture, respectively) (Figure 7). No *Beauveria* colonies were observed in untreated soil (data not shown). In the soil with higher humidity, CAs produced a greater mass of hyphae and more abundant sporulation. From 10 CAs inoculated on the surface of 1 g of soil, 6.4 × 10^4^ and 2.3 × 10^5^ CFU were obtained after 7 days of incubation on soil at 17% and 30% moisture, respectively. *Beauveria* concentration was 3.6 times greater at 30% soil moisture; each CA was able to produce around 2.0 × 10^4^ propagules.

### 3.4 Pathogenicity of conidia produced by AgR-F704 aggregates against black beetle

**Figure 8.**
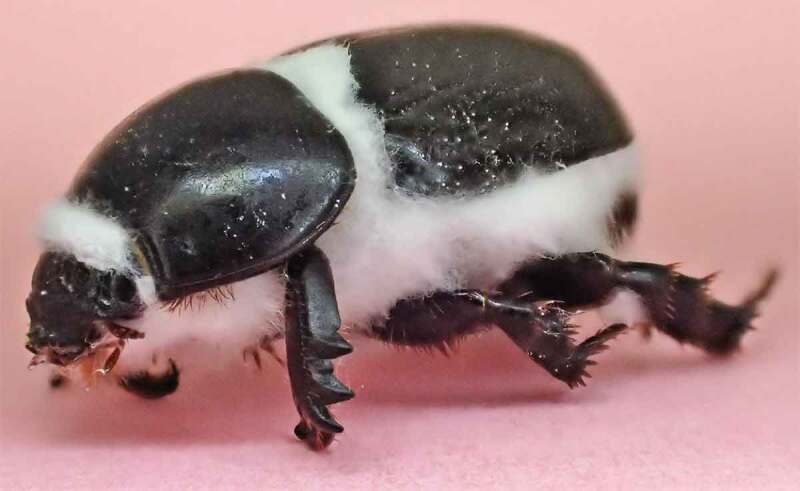
*H. arator* adult infected by conidia harvested from germinated CAs produced by *B. pseudobassiana* AgR-F704

The germination rate of conidia on water agar obtained from CAs produced by *B. pseudobassiana* was 98%. Treatment of these conidia against *H. arator* caused 51% mortality, which corresponds to 45% efficacy when corrected with the 13% mortality observed in the control treatment. Dead insects showed clear symptoms of *Beauveria* infection with white mycelium that emerged through the intersegmental spaces (Figure 8) and later spore abundantly beige, demonstrating that conidia produced by the CAs are infective propagules against *H. arator* adults.

## 4. Discussion

Aggregates formed by *B. pseudobassiana* AgR-F704 identified in the present study appear to have originated through the association of blastospores and conidia with a hyphal surface. The CAs grew through the aggregation of more free cells (predominantly blastospores) and hyphae fragments and also due to germination and growth of cells already trapped in the structure.

After 10 days of fermentation, CAs appeared as three-dimensional structures with a gelatinous texture and brownish colour. The structure of *B. pseudobassiana* CAs and their formation processes are similar to those described for biofilms, which are defined as complex surface-associated cell populations embedded in an extracellular matrix. Specific nutrients, quorum-sensing molecules, and surface contact are the principal signals for aggregation and cohesion of cells, together with the accumulation of an extracellular matrix as the biofilm matures, which seems to protect the cells against desiccation and toxic compounds (Nicholson and Moraes 1980; Fanning and Mitchell 2012).

Different environmental conditions have been reported as triggers for cell aggregation, in which diverse genetic factors and mechanisms are involved. For example, it has been suggested that oxidative stress, induced by factors including light, temperature, pH, and oxygen, could be related to MS formation in phytopathogenic fungi, as well as changes in iron and calcium cations that are closely related with MAPK signalling pathways (Song 2018). In our study, the fast agitation speed (300 rpm) used in our experiments could have triggered CA formation by causing an excessive oxygen concentration in the broth which could have induced oxidative stress during the fermentation process (Nagy 2002).

Alternatively, aggregation of *B. pseudobassiana* cells could have been triggered by electrostatic forces, hydrophobicity and interactions between spore wall components (Zhang and Zhang 2016). In this sense, the low pH in the culture medium used in the present work (pH 5.5) could have played an important role in promoting the aggregation. Fungal spores generally exhibit negative surface charges (Douglas et al. 1959) which are affected by pH and ionic strength. In this context, lower pH values are considered to cause positive charges which in turn increase spore aggregation (Zhang and Zhang 2016). It has been concluded that pH is the driving factor for electrostatic and hydrophobic interactions within fungal pellet/clump formation. Regarding interactions between spore wall components, no definite conclusion can be drawn from the scientific literature (Veiter et al. 2018). However, publications suggest that adhesion is mostly driven by the presence of polysaccharides (Zhang and Zhang 2016).

Entomopathogenic fungi can produce distinct cell types in liquid media, including submerged conidia and blastospores which can be distinguished by morphological and adhesive characteristics (Holder and Keyhani 2005; Cho et al. 2006). The type of cells forming the CAs was confirmed by an hydrophobicity test based on differences of affinity between blastospores and submerged conidia. Blastospores bind poorly to hydrophobic surfaces, but bind more readily to hydrophilic surfaces, while submerged conidia show broad binding characteristics being able to bind to both hydrophobic and hydrophilic surfaces (Holder and Keyhani 2005). Partition of cells released from the CAs into the two phases (aqueous and organic) and the presence of two cell morphotypes (spherical and ellipsoidal) confirmed that the CA structure contained both conidia and blastospores.

Cells forming the CAs were surrounded by an extracellular matrix similar to that observed in fungal biofilms, which provides a protective barrier from the surrounding environment (Mitchell et al. 2016). The composition of this extracellular matrix has been studied in some strains of *Saccharomyces cereviciae* that exhibit flocculation from clumping of cells or form surface-adherent biofilms. The extracellular matrix in those aggregates mainly contained glucose and mannose with a negligible amount of protein (Beauvais et al. 2009). Biofilms of *Aspergillus fumigatus* produced under *in vitro* and *in vivo* conditions that have been described as three-dimensional hyphal structures containing some conidia, also present an extracellular matrix, composed by exopolysaccharides, proteins, lipids and, interestingly, melanin (Mitchell et al. 2016), which was also observed in some CAs formed by the strain AgR-F704.

Blastospores are mainly formed when rich broth liquid cultures are used, while submerged conidia are produced under nutrient limitation conditions in the submerged cultures (Holder and Keyhani 2005). The culture media used in this study was a rich broth based on complex carbon sources and a C:N ratio of 5:1 (Villamizar et al. 2018), which induced an abundant production of blastospores and free hyphae for the first 4 days of culturing, accompanied by a low production of submerged conidia.

Aggregation of blastospores have previously been reported in yeasts such as *Candida albicans*, a commensal fungus that causes oral candidiasis in humans (Kumar et al. 2015). For *C. albicans*, formation of large cell-cell aggregates has been reported as mediated by the expression of a specific set of virulence factors that promote hyphal formation, adhesion and invasion of host tissues (Chaffin 2008; Kumar et al. 2015). Mature biofilms of *C. albicans* exhibit a more heterogenous structure, composed of blastospores and hyphae surrounded by an extracellular matrix of polysaccharide material which give a gel-like hydrated three-dimensional structure to the biofilm where the cells become partially immobilised (Cavalheiro and Teixeira 2018).

The strain AgR-F704 initiated CA formation after 2 days of liquid fermentation and structures looked melanised and mature after 10 days under the evaluated conditions, similar to the times previously reported for MS formation with the same strain (Villamizar et al. 2018) but longer than the time usually reported for fungal biofilm formation. For example, biofilm formation by the plant pathogen *Trichosporon asahii* occurs in an organised fashion through four distinct developmental phases: initial adherence of yeast cells (0 to 2 h), germination and microcolony formation (2 to 4 h), filamentation (4 to 6 h), and proliferation and maturation (24 to 72 h) (Di Bonaventura et al. 2006).

In our previous work, we described microsclerotia formation with three *Beauveria* species using liquid fermentation (Villamizar et al. 2018). However, *B. pseudobassiana* AgR-F704 behaved differently from the other two species (*Beauveria bassiana* and *Beauveria brogniartii*), showing greater blastospore production and smaller (<527 µm), less compact and less melanised structures after 10 days of fermentation. For this reason, the aggregates of *B. pseudobassiana* AgR-F704 were analysed in more detail in the present work. Using higher magnification and resolution to study the formation process and the structure of CAs from *B. pseudobassiana* AgR-F704, we found that they are distinct from MS, because are mainly formed by blastospores and conidia and not by hyphae. However, these CAs could have a similar function as microsclerotia acting as resistant structures, enabling fungi to survival and persist in harsh environments and being able to produce and release infective propagules in the field after periods of stress.

The yield of AgR-F704 CAs was in the order of 10^3^ CAs/mL after 10 days, similar to yields reported by Wang et al. (2011) for MS production with *B. bassiana* strains (6.2 x 10^2^–1.7 × 10^3^ MS/mL), but lower than those reached for MS production with other fungal species (>10^4^ MS/mL) over shorter time periods (Kobori et al. 2015; Song et al. 2016, 2017). However, no data is available in relation to the yield when fungal three-dimensional aggregates or biofilms are produced in artificial media.

CAs of *B. pseudobassiana* AgR-F704 showed accumulation of vacuolated cells, as was described for MS of *Verticillium dahliae* Kleb (Griffiths 1970) in which cells had undergone a process of autolysis coinciding with the accumulation of membranous material within the hyphae. Additionally, the presence of melanising particles was also observed in many cells within the CAs from AgR-F704. Melanin is a dark pigment produced by the oxidation of some aminoacids (Nosanchuk and Casadevall 2003), which provides defence against environmental stresses such as ultraviolet (UV) light, oxidising agents and ionising radiation. Melanin contributes to the ability of fungi to survive in harsh environments (Eisenman and Casadevall 2012).

The autolysis of cells during germination of CAs could be a mechanism to release nutrients to be used as energy sources for developing metabolic processes, which will be activated when conditions of humidity are favourable for germination. For the CAs obtained in the present work, some nutritional reserves are available in the extracellular matrix, but lysis of cells during germination could release different nutrients required for specific metabolic processes (Noor 2015). Autolysis has been demonstrated as an important process in biofils formation and maturation under starvation, when a part of cells inside the biofilm autolyse providing nutrients for the remaining cells. In addition, extracellular DNA, an essential component of the biofilm matrix, is released during biofilm development by cell lysis and contributes to the stability and development of the biofilm by binding to other biopolymers contained in the extracellular matrix. Moreover, through an acid–base interaction, it promotes adhesion between cells and between cells and surfaces (Allocati et al. 2015).

In our study, CAs produced by *B. pseudobassina* AgR-F704 exhibited excellent stability over 24 months at 4°C, maintaining the same myceliogenic germination capacity over the whole period of time. CAs also germinated and sporulated when they were incubated in wet soil, producing approximately 2.0 × 10^4^ propagules per each aggregate. This potential to produce infective conidia makes these structures a promising ingredient for use as a mycoinsecticide, which was confirmed by assessing its pathogenicity against black beetle adults. Conidia produced by CAs after germination and sporulation were able to infect and kill the insects as has also been demonstrated for conidia produced from germinating microsclerotia of *C. truncatum* and *M. anisopliae*, showing infectivity against their respective weed and insect hosts (Schisler and Jackson 1996; Jaronski and Jackson 2008). In other works, conidia (sporogenic germination) from MS of *Mycoleptodiscus terrestris* and *Purpureocillium lilacinum* were also capable of infecting and killing *Hydrilla verticillata* (Shearer and Jackson 2006) and the root-knot nematode, *Meloidogyne incognita* (Song et al. 2016), respectively.

In summary, we induced *B. pseudobassiana* to form three-dimensional cell aggregates in liquid fermentation, which formation and structure is consistent with the definition and description of fungal biofilms, which have never been reported before for *Beauveria* species. These CAs behave similar to fungal MS, exhibiting hyphal germination, abundant conidia production and pathogenicity against black beetle adults. All these properties suggest that the CA propagules can be used to develop a new biopesticide.

## 5. Conclusions

CAs produced by *B. pseudobassiana* AgR-F704 are aggregates or three-dimensional biofilms mainly formed by blastospores and conidia but containing few hyphae within the structure. Cells forming the CAs are surrounded by an extracellular matrix similar to that observed in fungal biofilms and have abundant crystals with unknown composition trapped inside. Autolysis of internal cells is produced during CA germination, which could be a mechanism to release nutrients to be used as energy sources to carry out metabolic processes. These CA structures are melanised and performed similarly to commonly described MS, being able to germinate and sporulate under adequate conditions of humidity, producing and releasing infective conidia with pathogenic activity against the African black beetle, *H. arator*. These results shed light on the potential use of CAs produced by *B. pseudobassiana* as a new delivery system of infective conidia to be used as active ingredient in novel biopesticides to control soil-dwelling insects.
